# Antimicrobial Efficacy of Endodontic Irrigants against *Enterococcus Faecalis* and *Escherichia Coli*: An *in vitro* study

**DOI:** 10.5005/jp-journals-10005-1214

**Published:** 2013-10-14

**Authors:** Noopur Kaushik, Usha Rehani, Abhay Agarwal, Mayur Kaushik, Vivek Adlakha

**Affiliations:** Senior Lecturer, Department of Pedodontics, Subharti Dental College Meerut, Uttar Pradesh, India, e-mail: drmayurkaushik@gmail.com; Professor, Department of Pedodontics, Inderprastha Dental College New Delhi, India; Professor, Department of Pedodontics, Kalka Dental College, Meerut Uttar Pradesh, India; Reader, Department of Periodontics, Subharti Dental College, Meerut Uttar Pradesh, India; Reader, Department of Pedodontics, Subharti Dental College, Meerut Uttar Pradesh, India

**Keywords:** Root canal irrigants, *Enterococcus faecalis*, *Escherichia Coli*, Cetrimide, Sodium hypochlorite

## Abstract

**Aim:** To evaluate the relative antimicrobial efficacy of five different commonly used antimicrobial agents with regard to reduction in the number of *Enterococcus faecalis* and *Escherichia coli* as compared to normal saline. An agar disk diffusion *in vitro* method was used to test the efficacy of the root canal irrigants against these two microorganisms. The root canal irrigants used were: 5.25% sodium hypochlorite (NaOCl), 10% citric acid, 17% ethylene diamine tetraacetic acid (EDTA), 3% hydrogen peroxide (H_2_O_2_), 0.2% cetrimide and normal saline (as control).

**Materials and methods:** The sample size consisted of 120 agar plates, divided into two groups: groups I and II. Group I consisted of 60 blood agar plates for assessment of *E. faecalis* and group II consisted of 60 MacConkey agar plates for assessment of *E. coli*. On each agar plate, 6 circular cellulose nitrate paper disks were placed, on which the inoculum of the respective microorganism was poured with a micropipette. After incubation, these paper disks were removed and put in test tubes containing the particular root canal irrigants, and were vortexed for 60 seconds.The microbial count was then assessed using a microbial colony counter.

**Results:** Results showed that in the group I (*E. faecalis*), maximum reduction was achieved with cetrimide, followed by NaOCl, H_2_O_2,_ citric acid and then EDTA. In group II (*E. coli*), maximum reduction was achieved with NaOCl, followed by cetrimide, H_2_O_2,_ citric acid and then EDTA.

**How to cite this article:** Kaushik N, Rehani U, Agarwal A, Kaushik M, Adlakha V. Antimicrobial Efficacy of Endodontic Irrigants against *Enterococcus Faecalis* and *Escherichia Coli*: An *in vitro* study. Int J Clin Pediatr Dent 2013;6(2):178-182.

## INTRODUCTION

Bacteria play the primary role in the development of necrotic pulps, periapical pathosis and post-treatment disease following root canal treatment. Elimination of microbes from the pulpal tissue as well as root canals is the main goal in order to prevent and treat pulpal and periapical breakdown. Successful root canal therapy relies on the combination of proper instrumentation, disinfection and obturation of root canal.^1^

Mechanical instrumentation is the core method for bacterial reduction in the infected root canal, but achieving bacteria-free root canals still proves to be difficult.^2^ Various studies have demonstrated that mechanical preparation with hand instruments and irrigation with saline cannot predictably eliminate the bacteria from the infected root canals.^3-5^ Therefore, the current focus of interest has been the use of irrigating solutions with strong antibacterial activity as the necessary supplement to mechanical preparation.

Over the years, various root canal irrigants have been used like sodium hypochlorite (NaOCl), hydrogen peroxide (H_2_O_2_), citric acid, cetrimide, ethylene diamine tetraacetic acid (EDTA), chlorhexidine. Out of these, sodium hypochlorite (NaOCl) is the most widely used irrigating solution.^6^

Even after meticulous mechanical procedures along with the use of irrigating solutions, some bacteria can still be recovered from the canals. The genera that most frequently persist include enterococci, staphylococci and Gram-negative enteric rods. *Enterococcus faecalis*, a Gram-positive facultative anaerobe, is commonly found in the root canals of failing endodontically treated cases.^7^ Another microorganism which has been often found in infected root canals is *Escherichia coli*,^8^ a Gram-negative facultative anaerobe, although research literature on this particular microorganism is sparse. The microorganisms found in the root canals of primary teeth are similar to those in the root canals of permanent teeth.^9^

The persistence of these microorganisms in the root canals, even after the use of irrigating solutions suggests that the choice of the solution used is largely determined still on empirical data. The answer of the ideal antimicrobial agent still eludes the clinician.

Thus, in the present study the relative antimicrobial efficacy of five different commonly used antimicrobial agents were compared with regard to reduction in the number of *Enterococcus faecalis* and *Escherichia coli* as compared to normal saline.

## MATERIALS AND METHODS (Table 1)

**Fig. 1:**
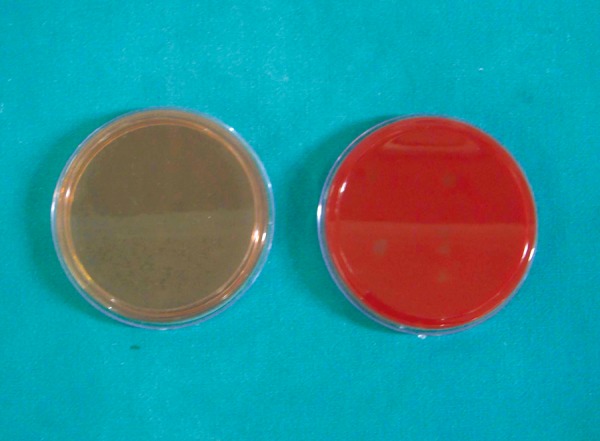
MacConkey's agar and blood agar plates

The sample size for this *in vitro* study consisted of 120 plates, which were divided into 2 groups of 60 plates each. Group I consisted of 60 plates of blood agar, used for evaluation of the reduction of *E. faecalis* in response to the test solutions. Group II consisted of 60 plates of MacConkey's agar (Fig. 1), used for evaluation of reduction of *E. coli* in response to the test solutions. Both the groups were further subdivided into 6 subgroups, according to the reagent used.

**Fig. 2:**
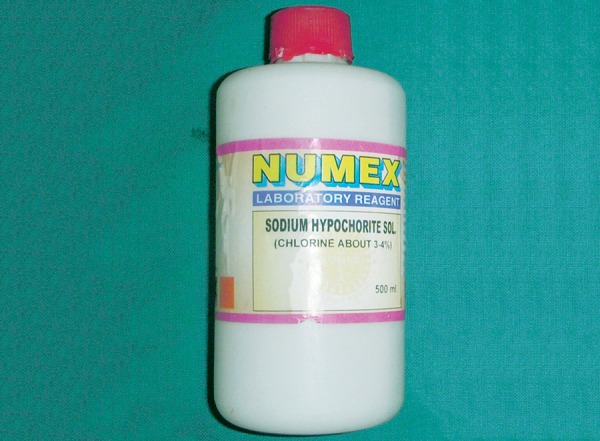
Sodium hypochlorite solution

Subgroup A : 5.25% sodium hypochlorite (NaOCl) (Fig. 2)

**Fig. 3:**
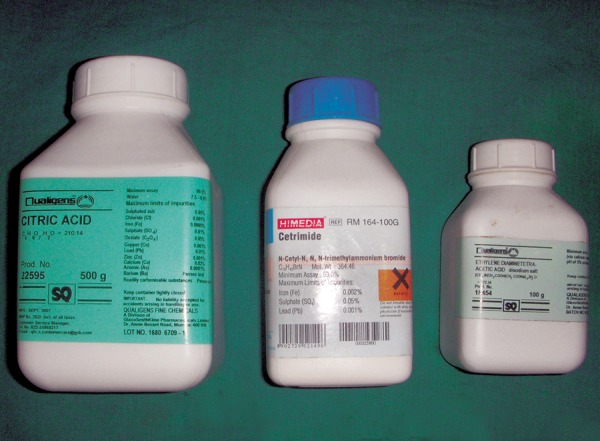
Citric acid, cetrimide and EDTA

Subgroup B : 10% citric acid (Fig. 3)

Subgroup C : 17% ethylene diamine tetraacetic acid (EDTA) (Fig. 3)

**Fig. 4:**
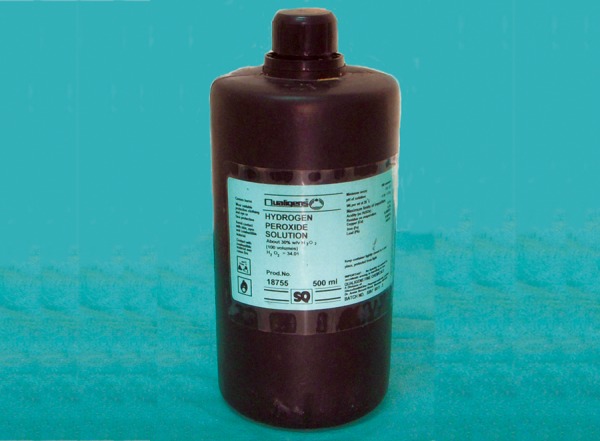
Hydrogen peroxide solution

Subgroup D : 3% hydrogen peroxide (H_2_O_2_) (Fig. 4)

Subgroup E : 0.2% cetrimide (Fig. 3)

**Fig. 5:**
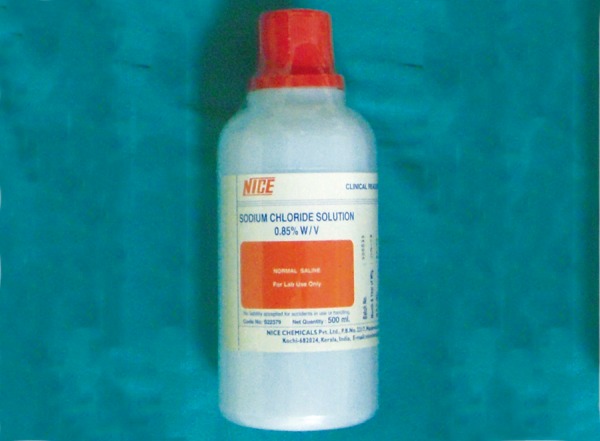
Sodium chloride solution

Subgroup F : Normal saline (NaCl) – as control group (Fig. 5)

**Table 1:** **Table 1:** Group distribution of different irrigating solutions

*Irrigating solution*		*Group I (E. faecalis)*		*Group II (E. coli)*
(A) 5.25% NaOCl		IA		IIA
(B) 10% citric acid		IB		IIB
(C) 17% EDTA		IC		IIC
(D) 3% H O		ID		IID

**Fig. 6:**
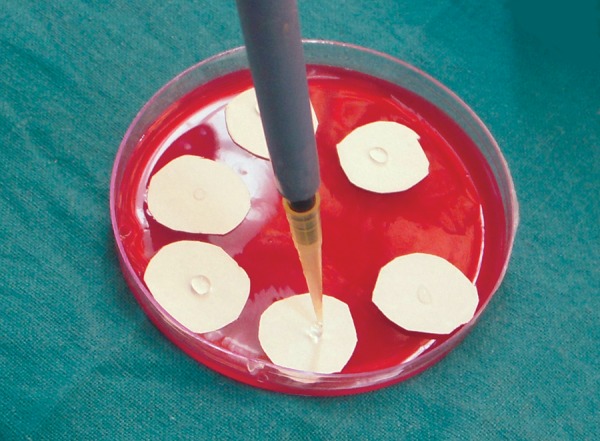
Bacterial broth placed on cellulose nitrate paper

**Fig. 7:**
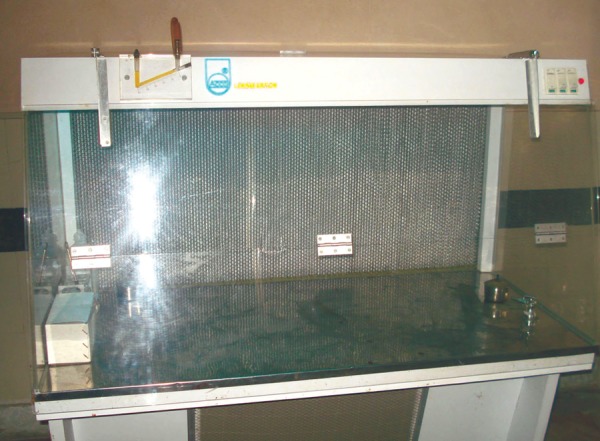
Laminar flow cabinet

**Fig. 8:**
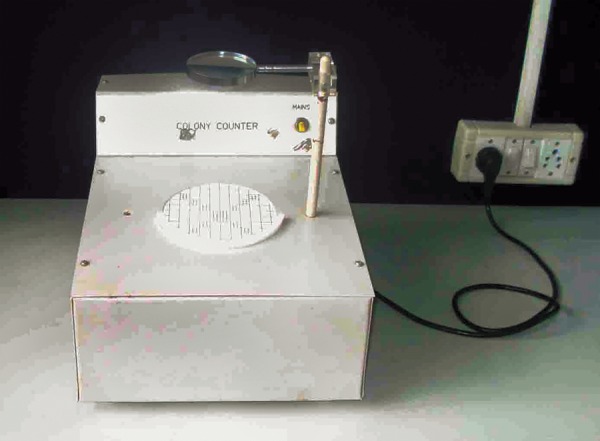
Microbial colony counter

Overnight cultures of *E. faecalis* (ATCC 29212) and *E. coli* (ATCC 25922) were obtained in broth form. Six circular discs of 2 cm diameter of sterile cellulose nitrate paper were put in each of the blood agar and MacConkey's agar plates. 60 μl each of *E. faecalis* and *E. coli* broth were taken with the help of micropipettes (Fig. 6). All the procedures were carried out in a laminar flow cabinet (Fig. 7) to maintain the sterility of the samples. After 24 hours, the filter paper disks were removed from each plate and put in six separate test tubes containing 2 ml each of the 6 reagents for 5 minutes. The test tubes were then vortexed for 60 seconds each. Serial dilutions were made for each of the samples. 10 μl was taken from each of the test tubes and spread on separate blood agar plates and MacConkey's agar and incubated for 24 hours. After 24 hours, the blood agar and MacConkey's agar plates were shifted to a microbial colony counter (Fig. 8) to estimate the relative reduction of the microorganisms.

**Table 2:** **Table 2:** The reduction of *E. faecalis* in response to the various irrigants (group I)

*Group IA 5.25% NaOCl*		*Group IB 10% citric acid*		*Group IC 17% EDTA*		*Group ID 3% H_2_O_2_*		*Group IE 0.2% cetrimide*		*Group IF Saline (control)*
12 × 102		25 × 105		36 × 107		18 × 104		5 × 101		43 × 109
10 × 10^2^		11 × 10^5^		22 × 10^7^		16 × 104		2 × 10^1^		39 × 10^9^
12 × 10^2^		17 × 10^5^		35 × 10^7^		18 × 10^4^		1 × 10^1^		42 × 10^9^
7 × 10^2^		21 × 10^5^		11 × 10^7^		12 × 10^4^		7 × 10^1^		15 × 10^9^
9 × 10^2^		19 × 10^5^		17 × 10^7^		15 × 10^4^		6 × 10^1^		37 × 10^9^
15 × 10^2^		9 × 10^5^		19 × 10^7^		11 × 10^4^		0		17 × 10^9^
10 × 10^2^		14 × 10^5^		13 × 10^7^		14 × 10^4^		8 × 10^1^		9 × 10^9^
11 × 10^2^		23 × 10^5^		32 × 10^7^		20 × 10^4^		6 × 10^1^		45 × 10^9^
12 × 10^2^		12 × 10^5^		9 × 10^7^		13 × 10^4^		0		33 × 10^9^
8 × 10^2^		17 × 10^5^		27 × 10^7^		12 × 10^4^		4 × 10^1^		21 × 10^9^

**Table 3:** **Table 3:** The reduction of *E. coli* in response to the various irrigants (group II)

*Group IIA 5.25% NaOCl*		*Group IIB 10% citric acid*		*Group IIC 17% EDTA*		*Group IID 3% H_2_O_2_*		*Group IIE 0.2% cetrimide*		*Group IIF Saline (control)*
3 × 10^1^		25 × 10^5^		35 × 10^7^		21 × 10^4^		15 × 10^2^		45 × 10^9^
5 × 10^1^		21 × 10^5^		37 × 10^7^		9 × 10^4^		17 × 10^2^		29 × 10^9^
7 × 10^1^		29 × 10^5^		29 × 10^7^		23 × 10^4^		11 × 10^2^		41 × 10^9^
2 × 10^1^		14 × 10^5^		33 × 10^7^		17 × 10^4^		15 × 10^2^		52 × 10^9^
9 × 10^1^		17 × 10^5^		21 × 10^7^		19 × 10^4^		9 × 10^2^		23 × 10^9^
7 × 10^1^		22 × 10^5^		11 × 10^7^		13 × 10^4^		7 × 10^2^		31 × 10^9^
11 × 10^1^		9 × 10^5^		27 × 10^7^		14 × 10^4^		11 × 10^2^		37 × 10^9^
3 × 10^1^		19 × 10^5^		16 × 10^7^		25 × 10^4^		13 × 10^2^		19 × 10^9^
6 × 10^1^		26 × 10^5^		39 × 10^7^		20 × 10^4^		17 × 10^2^		46 × 10^9^
5 × 10^1^		25 × 10^5^		25 × 10^7^		12 × 10^4^		10 × 10^2^		47 × 10^9^

## OBSERVATIONS AND RESULTS

**Graph 1:**
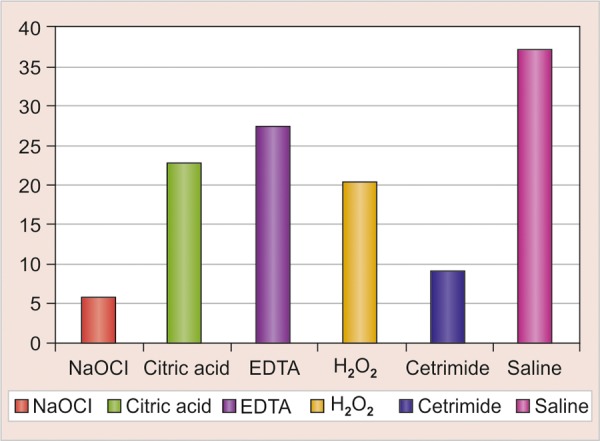
Comparative efficacy of reagents against *E. faecalis*

The results obtained were analyzed using one-way ANOVA (F-test). The results were further analyzed comparing two subgroups at one time using unpaired ‘t’ test. This test is used to find whether statistically significant difference is there between two individual subgroups or not. In group I, cetrimide produced the maximum reduction in number of *E. faecalis* (Graph 1 and Table 2).

**Graph 2:**
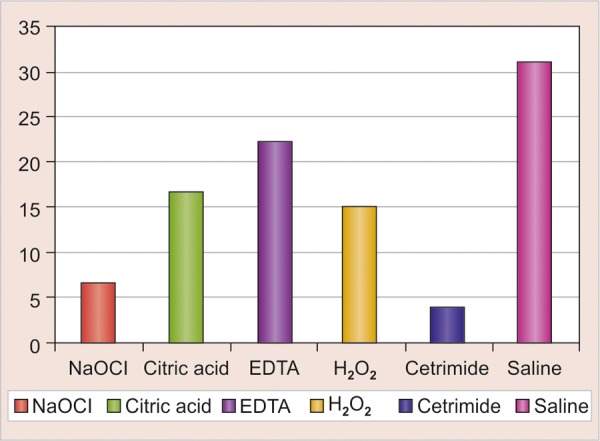
Comparative efficacy of reagents against *E. coli*

In group II, NaOCl showed maximum reduction in number of *E. coli* (Graph 2 and Table 3).

In both the groups, all the tested reagents showed significant reduction in bacterial count as compared to saline (control). NaOCl and cetrimide showed significant reduction as compared to H_2_O_2_, cetrimide and EDTA. The reduction was insignificant between NaOCl and cetrimide. The reduction was insignificant between H_2_O_2_, cetrimide and EDTA.

## DISCUSSION

Failure of endodontic treatment can be attributed to the fact that mechanical instrumentation alone does not eliminate the bacterial load completely. Thus, the use of antimicrobial agents, as adjuncts for irrigation and medication of root canals, has been shown to help reduce the bacterial counts further.^2^

In the current study, 5.25% NaOCl was used as an irrigant to test its efficacy against *E. faecalis* and *E. coli* and it proved to be a useful root canal irrigant.

The high success rate in microbial reduction with NaOCl in the present study, are in accordance with the studies.^2,8,10-13^

The efficacy of NaOCl was statistically more significant than HO, citric acid and EDTA in both *E. faecalis* and *E. coli* groups. The higher success rate with NaOCl can be attributed to its double action, i.e. it causes dissolution of necrotic tissues (due to its high pH) and is also germicidal.^14^

With regard to cetrimide, in group I (*E. faecalis*), cetrimide performed better than NaOCl in microbial reduction. However, in group II (*E. coli*), it showed lesser antimicrobial effect than NaOCl, although the difference was not statistically significant. The reagent which produced the maximum microbial reduction, after NaOCl and cetrimide, in both the groups, was Hydrogen peroxide (HO). Hydrogen peroxide demonstrates broad spectrum efficacy against viruses, bacteria, yeasts and bacterial spores. The results of the present study are in conjunction with the study done by Naenni et al,^13^ which showed that HO was superior to citric acid but inferior to NaOCl as a root canal irrigant.

Citric acid produced the maximum microbial reduction in both the groups, after NaOCl, cetrimide and HO. It is proven that citric acid is effective in removing the smear layer in concentrations of 10, 25 and 50%.^10,15^ EDTA produced the least reduction in the microbial count in both the groups. These results are in accordance with the study done by Moliz et al,^16^ which showed that EDTA and citric acid were not effective against the biofilms of *E.faecalis* at any concentration or time tested.

The uniqueness of the present study lies in the study design in which five of the most commonly used root canal irrigants have been tested simultaneously. Also, this is one of the few studies^8,14,17^ which have been done with regard to the persistence of *E. coli* in response to root canal irrigants. Finally, the results of the present study question the use of sodium hypochlorite as the most effective root canal irrigant since cetrimide provided comparable results to sodium hypochlorite.

## SUMMARY AND CONCLUSION

From this study, the following conclusions can be drawn:

1. Cetrimide showed the maximum reduction in the number of microorganisms in group I (*E. faecalis*) followed by NaOCl.
2. Hydrogen peroxide was the next best reagent followed by citric acid and EDTA in both the groups.
3. Sodium hypochlorite showed the maximum reduction in the number of microorganisms in group II (*E. coli*). The limitations of the present study include:
  1. A clinical *in vivo* study could have been done in conjunction with the *in vitro* study, to prove the clinical effectiveness of these endodontic irrigants.
  2. Pure biofilms were used of *E. faecalis* and *E. coli* separately to test the efficacy of the irrigants. Studies should be done focusing on polymicrobial biofilms, rather than individual microorganisms. These considerations could be a future avenue of research with regard to endodontic studies on root canal irrigants.
